# Lavage with Allicin in Combination with Vancomycin Inhibits Biofilm Formation by *Staphylococcus epidermidis* in a Rabbit Model of Prosthetic Joint Infection

**DOI:** 10.1371/journal.pone.0102760

**Published:** 2014-07-15

**Authors:** Haohan Zhai, Jianchao Pan, En Pang, Bo Bai

**Affiliations:** 1 Department of Orthopaedic Surgery, the First Affiliated Hospital of Guangzhou Medical University, Guangzhou, China; 2 Department of Microbiology, Kingmed Diagnostic Center Co. Ltd, Guangzhou, China; Columbia University, College of Physicians and Surgeons, United States of America

## Abstract

**Background and Aim:**

The present anti-infection strategy for prosthetic joint infections (PJI) includes the use of antibiotics and surgical treatments, but the bacterial eradication rates are still low. One of the major challenges is the formation of biofilm causing poor bacterial eradication. Recently it has been reported that allicin (diallyl thiosulphinate), an antibacterial principle of garlic, can inhibit bacteria adherence and prevent biofilm formation *in vitro*. However, whether allicin could inhibit biofilm formation *in vivo* is unknown. The aim of this study was to investigate the effects of allicin on biofilm formation, and whether allicin could potentiate the bactericidal effect of vancomycin in a rabbit PJI model.

**Methods:**

A sterile stainless-steel screw with a sterile ultra-high molecular weight polyethylene washer was inserted into the lateral femoral condyle of the right hind knee joint of rabbit, and 1 mL inoculum containing 10^4^ colony-forming units of *Staphylococcus epidermidis was* inoculated into the knee joint (n = 32). Fourteen days later, rabbits randomly received one of the following 4 treatments using continuous lavages: normal saline, vancomycin (20 mcg/mL), allicin (4 mg/L), or allicin (4 mg/L) plus vancomycin (20 mcg/mL). Three days later, the washer surface biofilm formation was examined by scanning electron microscopy (SEM). The bacterial counts within the biofilm of implanted screws were determined by bacterial culture.

**Results:**

The lowest number of viable bacterial counts of *Staphylococcus epidermidis* recovered from the biofilm was in the rabbits treated with allicin plus vancomycin (P<0.01 vs. all other groups). The biofilm formation was significantly reduced or undetectable by SEM in rabbits receiving allicin or allicin plus vancomycin.

**Conclusion:**

Intra-articular allicincan inhibit biofilm formation and enhance the bactericidal effect of vancomycin on implant surface *in vivo*. Allicin in combination with vancomycin may be a useful anti-infection strategy for the treatment of PJI.

## Introduction

Prosthetic joint infection (PJI) is a rare, but one of the most devastating complications in total joint arthroplasty [1], [2], [3], that an arthritic or dysfunctional joint is removed and replaced with an artificial joint. Approximately 1–2% of the patients who undergo prosthetic joint arthroplasty develop PJI in the first 2 years after the surgery [4]. As arthroplasty becomes more common, more patients are expected to suffer from PJI [3]. PJI causes poor function of the prosthetic joint, requires long-term use of antibiotics and even multiple surgical procedures, which impose a substantial economic burden on the individuals and the health care system [3], [5], [6]. Unfortunately, the treatment of PJI is extremely difficult. Previous studies indicated poor bacterial eradication rates [7], [8], [9]. The eradication rates were 18% to 37% for methicillin-resistant *Staphylococcus aureus* (MRSA) [7], [8], and 65% and 75% for streptococcal infections and all other organisms [9], respectively. Some analyses regarded staphylococcal infection as an independent predictor of failed treatment relating to irrigation and debridement with implant retention [10]. The success rates for treating PJI caused by a gram-negative pathogen, MRSA, and methicillin-sensitive gram-positive organisms were 52%, 51% and 69%, respectively, even with a two-stage procedure [11] For antibiotics to achieve satisfying results, it is important that the activity of antibiotic is preserved in the presence of biofilm [6]. There are no standard length of the initial intravenous treatment and the total treatment duration [12]. In addition, there are significant differences of practices in North America, Europe and Australia [13]. The optimal treatment protocol of PJI is still under intense investigation and open to debate [6], [14]. A majority of prosthesis-related infections are caused by *Staphylococcus epidermidis* (*S. epidermidis*) and *Staphylococcus aureus* [12], [15]. Polyethylene seems to be strongly attractive for *S. epidermidis*, while prosthetic metals are more suited for *Staphylococcus aureus* adherence [15]. Therefore, multiple factors are responsible for the differences in the eradication rates in the treatment of PJI. Biofilm produced by planktonic bacteria adhering to the implants makes the treatment of PJI more difficult [12]. Biofilm can resist host cellular and humoral immune responses, and bacteria within the biofilm are less susceptible to antimicrobial agents [16], [17]. Biofilm formation and development are a dynamic process that includes attachment, biofilm formation, and dispersal [18]. Bacteria within the biofilm can remain in the body for a prolonged period of time, and become a source of bacteria that disseminate to other sites of the implant surface, forming new biofilms. In conventional antibiotic therapy, some of planktonic bacteria are killed, while others quickly form new biofilms on the implant; this latter observation may explain the chronic nature of most implant device infections [19]. So inhibiting planktonic bacteria adhering to implant and biofilm formation is a potentially useful strategy to eradicate biofilm-related prosthesis infections [20].

Garlic has been used to treat infections since ancient times. Its active ingredient is allicin. Allicin is an antibacterial agent with *in vitro* activity against biofilm formation of *S. epidermidis.* It has been demonstrated that sub-MIC concentration of allicin may prevent *S. epidermidis* adherence and biofilm formation *in vitro* [21], [22]. Biofilm detachment release single cells or larger cell clusters, which prompts the dispersal of bacteria to other sites and formation new biofilms [23]. Vancomycin is the treatment of choice for MRSA [24] and *S. epidermidis* infections [25]. However, vancomycin alone is ineffective in eradicating staphylococcal infection on orthopaedic device [12], [25]; its combination with rifampin is recommended to treat PJI caused by MRSA or *S.epidermidis* [12], [25]. Since allicin can prevent bacteria adhesion and aggregation, and inhibit biofilm formation *in vitro*, we therefore hypothesized that the vancomycin bactericidal effect can be enhanced on the implant surface by co-administration of allicin. In this study, using a rabbit model of PJI caused by *S. epidermidis,* we investigated the effect of intra-articular lavage of a combination of allicin and vancomycin on prevention of new biofilm formation and elimination of prosthesis surface bacteria.

## Materials and Methods

### Bacterial strain

*Staphylococcus epidermidis* strain *RP62A* (a biofilm-forming bacteria [26]) was obtained from ATCC (Catalog number 35984) and propagated in tryptic soy broth media.

### Antimicrobial Agents

The normal saline lavage solution contained vancomycin (Eli Lilly, Kobe, Japan) and/or allicin (RYEN Pharma, Jiangsu, China). The concentration of vancomycin in the lavage fluid was 20 mcg/mL; this concentration was selected because acceptable trough concentrations of vancomycin are generally between 10 and 20 mcg/mL [24]. The concentration of allicin in the lavage fluid was 4 mg/L; this concentration was chosen as it has been previously shown to prevent *S. epidermidis* biofilm formation [21].

### Rabbit PJI model

All animal experiments were approved by the Animal Care and Ethics Committee of Guangzhou Medical University (Permit Number: 2013-92). Thirty-two New Zealand White rabbits (weight: 2.5–3.5 kg) were used. The rabbits were housed separately in individual cages. They were exposed to a natural light-dark cycle and given free access to food and water.

The rabbit PJI model was previously described [27] with minor modifications. The amount of bacteria inoculation of *S. epidermidis* was pre-determined by pre-inoculation. Briefly, 32 New Zealand rabbits were randomly divided into 4 groups (8 in each group). A sterile stainless-steel screw and a sterile ultra-high molecular weight polyethylene (UHMWPE) washer were implanted in the rabbit knee joint. A titration of 10^3^, 10^4^, and 10^5^ CFU *S. epidermidis* (strain *RP62A*) and 1 mL normal saline (as a negative control) were inoculated into the knee joint of each group. After two weeks, synovium and Prosthesis eluent were cultured for bacteria to determine joint infection. The rabbits with knee infection were 0 (saline group), 6 (10^3^ CFU group), 8 (10^4^ CFU group), 8 group (10^5^ CFU). There were 2 rabbits with poor wound healing, and both were in the 10^5^ CFU group. There were a large amount of biofilms on UHMWPE washer surface detected by SEM in the 10^4^ CFU group, and the *S. epidermidis* grew and aggregated within the biofilms. Hence, we determined that 1 mL 10^4^ CFU *S. epidermidis* was the appropriate dose to generate knee infection model.

Surgery was performed under general anesthesia with intramuscular injection of ketamine (35 mg/kg) and diazepam (5 mg/kg) as a single dose. After cutting the hairs in the area, a 2 cm longitudinal skin incision was made along the lateral aspect of the right hind knee joint. The fascia and joint capsule were then incised, exposing the lateral femoral condyle and the lateral collateral ligament (LCL). A hole was drilled (with a 3.5 mm sterile drill bit) into the lateral femoral condyle, anterior to the LCL and in the direction of the coronal axis. A sterile stainless-steel screw (3.5 mm diameter, 15 mm length) with a sterile UHMWPE washer (3.5 mm inner diameter, 8 mm external diameter, 1.5 mm thickness) was inserted into the hole. The joint capsule, deep fascia and skin were closed with 4–0 nylon suture. Immediately after surgery, 1 mL inoculum containing 10^4^ colony-forming units (CFUs) of *S. epidermidis* was inoculated into the knee joint, near the implants. Each rabbit received acetaminophen 30 mg/kg/day for analgesia for 3 days following surgery. We have recorded the temperature and weight of the animal, and not found the rabbits to have fever or weight loss.

### Study groups and intra-articular lavage

Fourteen days after bacteria inoculation, rabbits were randomly assigned to four groups of eight rabbits each, and were given continuous intra-articular lavage of the following solutions: normal saline (Group A); vancomycin 20 mcg/mL (Group B); allicin 4 mg/L (Group C); and vancomycin 20 mcg/mL plus allicin 4 mg/L (Group D).

The intra-articular lavage device was installed as follows. Under general anesthesia as mentioned above, a 2.5 cm skin incision was made in the midline of the knee. A 12-gauge needle was inserted into the joint cavity at the lateral suprapatellar port and then withdrawn. An intravenous infusion tubing was inserted through the channel formed by the 12-gauge needle until the tip was 1.5–2 cm into the knee joint cavity. It was secured in this position by suturing to the skin with 4-0 nylon suture. This tubing acted as the inflow conduit for the lavage. The outflow for the lavage was another intravenous infusion tubing, which was placed 1.5–2 cm into the knee joint at the medial infrapatellar area, using the same method as for the inflow tubing. This tubing was likewise fixed to the skin with 4-0 nylon suture. A plaster cast was applied to immobilize the knee joint. After recovery from anesthesia, the rabbits were housed in narrow cages that limited movement to prevent kinking (and thus blockage) of the lavage tubing. A 500 mL bag of lavage liquid was placed 1 meter above the knee and connected to the inflow tubing. Lavage fluids were administered continuously at 1–1.5mL/min for 3 days.

### Determination of biofilm formation and bacterial burden

The rabbits were euthanized with intravenous injection of phenobarbital 100 mg/kg 12 hours after completion of the lavage. The implanted screws and washers were harvested from the surgical site using sterile technique. The screws were rinsed three times with 5 mL phosphate-buffered saline (PBS) to remove surface planktonic bacteria, and then placed in a glass beaker, to which 5 mL sterile saline was added. The saline in the beaker was subsequently sonicated in an ultrasonic cleaner (Kunshan Hechuang Ultrasonic Co. Ltd, Jiangsu, China) using the procedure previously described [28]. One milliliter of the solution after sonication was used for bacterial culture. Samples were serially diluted in TSB and inoculated on TSB agar plates. After incubation at 37°C for 24 h, the final bacterial counts were calculated, and expressed as log_10_ colony forming unit (CFU)/mL. The experiments were repeated three times.

The washers were also rinsed three times with 5 mL PBS. Each washer was then preserved in fixative (2.5% glutaraldehyde) for 24 h, as described in previous study [29]. After dehydration and coating with gold–palladium, an observation area was randomly selected and marked on the washer. And then, the washers were analyzed with a scanning electron microscope (SEM) (JEOL Ltd., Tokyo, Japan). Because of their irregular shape, the screws cannot be scanned by SEM.

All individuals involved in the microbial experiments and SEM study were blinded to the identities of the samples.

### Statistical analyses

SPSS 17.0 (SPSS, Inc., Chicago, IL) software was used for the statistical analyses. Data are expressed as mean ± standard deviation (SD). The differences in bacterial burden of the biofilm among the groups were assessed by one-way analysis of variance (ANOVA) with the least significant difference (LSD) method. *P*<0.05 was considered statistically significant.

## Results

**Figure 1:**
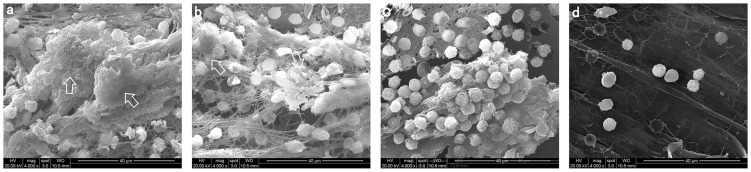
SEM images of biofilms and attached *Staphylococcus epidermidis* on the implant washers. Arrows indicate *Staphylococcus epidermidis* biofilm on the washers. Representative SEM images from animals received intra-articular lavage with normal saline (a), vancomycin (b), allicin (c) and allicin plus vancomycin (d) are shown. The magnifications are X1000for all images.

In the rabbit knee PJI model, we evaluated the effects of the antibiotics intra-articular lavage on biofilm formation and bacterial counts (*S. epidermidis*) on the prosthesis joints. SEM was used to directly visualize the biofilm formation and bacteria accumulation on the prosthesis washers in randomly selected areas (Figure 1). In the saline group (Figure 1 a), large amount of biofilms were seen and bacteria were densely packed. In the vancomycin group, biofilms were abundant and some bacteria were seen inlaid in the biofilms (Figure 1 b). In the allicin group, the biofilm formation was significant reduced and bacteria were still detectable (Figure 1 c). However, there were minimal or no biofilms visualized and only sporadic bacteria were detected on the washers in the group treated with both vancomycin and allicin intra-articular lavage (Figure 1 d), which were clearly fewer than those observed for the other groups.

**Figure 2:**
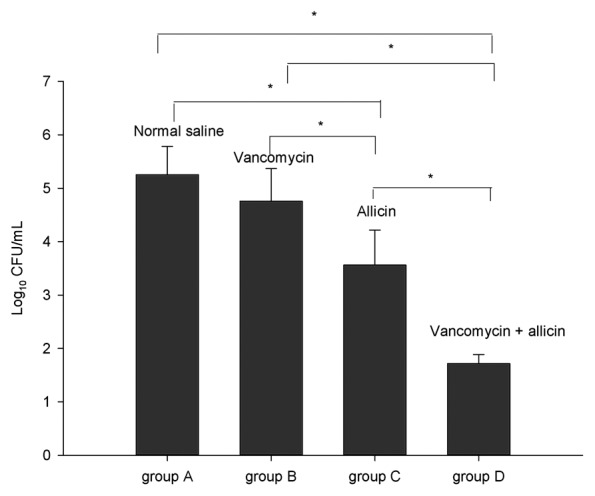
Bacterial counts of eluates from the knee joint implants (screw). Thirty two rabbits were randomly assigned to four groups of eight rabbits each. The viable organisms recovered from the screw eluate of the indicated groups are expressed as log_10_CFU/mL. The experiments of bacterial counts were repeated three times. The data are expressed as the mean and the error bars represent the standard deviation. Asterisks denote statistically significant differences: * P<0.01.

The bacteria detected by SEM was further verified and quantified by bacterial cultures. The bacterial counts of the sonicated eluates from the screws for each group are shown in Figure 2. There was a significant overall difference among the groups by ANOVA (F = 71.14, *P*<0.001). The bacterial counts (as expressed as log_10_ CFU/mL) of sonicated eluates of saline (group A), vancomycin (group B), allicin (group C), and vancomycin and allicin group (group D) were 5.25±0.53, 4.75±0.61, 3.56±0.66, and 1.71±0.19, respectively. The lowest bacterial count was observed in vancomycin plus allicin group which was significantly lower than all other groups (P<0.01). The bacterial count of sonicated eluate from the allicin group was slightly lower than the saline and vancomycin groups (P<0.01). The bacterial count difference between the vancomycin and saline group was not significant (P>0.05).

## Discussion

Biofilm infection is a potentially catastrophic complication of prosthetic joint replacement. Biofilm-embedded bacteria are 100–1000 times more resistant to antibiotics than their planktonic counterparts [16]. To improve the bactericidal effect of antimicrobial agents on biofilm-embedded bacteria, inhibition of bacteria adherence and biofilm formation are useful strategies. Recently, studies have shown that allicin can inhibit bacterial adhesion and aggregation, and then prevent the biofilm formation *in vitro* [21], [22]. In addition, the effect of allicin plus vancomycin on biofilm-related PJI *in vivo* has not been studied. In the present study, we evaluated the efficacy of intra-articular lavage using a combination of allicin and vancomycin for the treatment of PJI in a rabbit model. Our results showed that allicin inhibited the formation of biofilm on prosthetic joint surfaces *in vivo*; and combined use of allicin and vancomycin effectively killed bacteria within the biofilm.

Compared with normal saline, lavage with vancomycin plus allicin had strong bactericidal effects on biofilm-embedded bacteria of the screw surface. The rabbits received combination lavage had lower bacterial counts in cultures of the eluates of the screws implanted and no biofilm visualized by SEM on the washers. There are several possible explanations for this result, which are not mutually exclusive. Firstly, biofilm formation and development are dynamic processes [18], [30]. Bacteria released from biofilm dispersal adhere to the surface of implants and form new biofilms. In the presence of allicin, the biofilm-released bacteria (i.e. *S. epidermidis* in our study) are prevented to adhere and aggregate on the implanted materials surface in the articular cavity [21], and new biofilm formation is inhibited. The bacteria dissociate in the articular cavity and are more easily killed by antibiotics (vancomycin in our study). Secondly, the antimicrobials are only able to diffuse through the biofilm shallowly and kill bacteria located shallow in the biofilms [31]. In the presence of allicin, the new biofilm cannot form, and bacteria that are previously deep in biofilm and inaccessible to antibiotics are now exposed and rendered sensitive to antibiotics. In the SEM images, biofilm was significantly reduced or eliminated in the allicin-containing lavage groups (Figure 1 c, d). This provides *in vivo* evidence to support *in vitro* findings that allicin inhibits biofilm formation [21], [22]. The bacterial count in the sonicated eluates in the allicin group was slightly lower than that in the saline and vancomycin group (Figure 2). This is likely due to allicin preventing bacteria to form new biofilms, which leads to the decrease of residual biofilm-embedded bacteria on the prosthesis. The bacterial counts of the sonicated eluates in the vancomycin group were only slightly lower than that in the saline group (P>0.05). This suggests that vancomycin has poor bactericidal effect on biofilm-embedded bacteria. The shorter course of vancomycin treatment (3 days) might also be one of the contributing factors.

The mechanism(s) of biofilm formation is complex and the mechanism by which allicin inhibits biofilm formation is still poorly understood. Nevertheless, our results have shown, for the first time, that allicin inhibits *S. epidermidis* biofilm formation *in vivo*, an effect that was previously demonstrated only *in vitro*.

Intra-articular lavage with allicin plus vancomycin may be a useful anti-infection strategy for the treatment of PJI. Biofilm plays an important role in PJI and the successful treatment of PJI must eradicate the biofilm dwelling microorganisms [6], [13]. Debridement with retention of device is the most conservative surgical approach. The success rate was approximately 80% of appropriately selected cases [32], which was less than the single-stage and two-stage exchange procedures [12]. In implant-associated infection, the cure rate was extremely low by antibiotics alone, because even therapy with susceptible antibiotics cannot eliminate the biofilm from the surface of an implanted device [12]. The intra-articular minimal inhibitory concentration (MIC) is difficult to achieve by systemic antibiotics delivery [33]. By using of lavage, the intra-articular antibiotics concentrations can be insured. We speculate that allicin plus vancomycin lavage therapy in addition to the treatment of retained prosthesis could improve the cure rate of PJI.

In theory, longer lavage time could eliminate the residuals of biofilm-embedded bacteria and biofilms on the surface of the implant. However, longer lavage time could potentially lead to greater risk of infection. A balance of efficacy and risk needs to be sought. It has been demonstrated that the irrigation-drainage system was recommended for a period from 2 to 6 days [34]. Based on the study, 3 days was used as the lavage time in our study.

Joint lavage can wash out microscopic and macroscopic intra-articular debris from inside the joint space. It is commonly used in the treatment of knee osteoarthritis, septic arthritis, and temporomandibular joint disorders [35], [36], [37], [38], [39]. It has also been explored to treat PJI using different drugs in the lavage [40], [41], [42], [43], [44]. The continuous lavage can effectively reduce the bacterial load on various surfaces [45] and maintain the concentration of drug in the joint cavity. The results of our study support this notion.

A low dose of 10^4^ CUF *S. epidermidis* was inoculated into rabbit articular cavity, the SEM results presented a large of biofilm on prosthesis in the saline lavage group. We believe the real clinical situation of PJI was reflected by inoculating this amount of bacteria. If a higher dose of bacteria was inoculated, it might cause wound disunion, sinus formation, and other complications.

The animal model presented in this study replicated the intra-articular implantation of biomaterials commonly employed in joint prosthesis, and not the mechanics of human prosthetic joint replacement. This animal model is suitable for studying the effect of drugs on PJI, but not suitable for other artificial joint research, such as joint prosthesis loosening. Nevertheless, developing artificial joints animal models that more closely mimicking human prosthesis is necessary.

Further studies are needed to determine whether allicin can inhibit the biofilm produced by other bacteria. In this study, the biofilm on the implant surface was difficult to quantify. Because of the irregular shape, screws cannot be scanned by SEM. Further experiments should improve the metal implant shape for optimal observation by SEM. Future work should also include systemic administration of allicin combined with vancomycin to verify their synergistic effect in the treatment of PJI. In fact, the systemic administration has less risk regarding to exogenous infection and can be used for longer time than lavage.

In summary, our results showed allicin can inhibit *S. epidermidis* biofilm formation *in vivo* that has been previously demonstrated only *in vitro*. Combined with allicin, the bactericidal effect of vancomycin is enhanced. Allicin combined with vancomycin may thus be a useful anti-infection strategy for the retention prosthesis treatment of PJI.
